# Ferumoxytol-enhanced magnetic resonance imaging in acute myocarditis

**DOI:** 10.1136/heartjnl-2017-311688

**Published:** 2017-10-06

**Authors:** Colin G Stirrat, Shirjel R Alam, Thomas J MacGillivray, Calum D Gray, Marc R Dweck, Kevin Dibb, Nick Spath, John R Payne, Sanjay K Prasad, Roy S Gardner, Saeed Mirsadraee, Peter A Henriksen, Scott IK Semple, David E Newby

**Affiliations:** 1 British Heart Foundation/University Centre for Cardiovascular Science, University of Edinburgh, Edinburgh, UK; 2 Clinical Research Facility, University of Edinburgh, Edinburgh, UK; 3 Edinburgh Imaging QMRI Facility, University of Edinburgh, Edinburgh, UK; 4 Department of Cardiology, Golden Jubilee National Hospital, Clydebank, UK; 5 Department of Cardiology, Royal Brompton Hospital, London, UK; 6 Department of Radiology, Royal Brompton Hospital, London, UK

**Keywords:** cardiac, MRI, myocarditis, inflammation, USPIO.

## Abstract

**Objectives:**

Ultrasmall superparamagnetic particles of iron oxide (USPIO)-enhanced MRI can detect tissue-resident macrophage activity and identify cellular inflammation within tissues. We hypothesised that USPIO-enhanced MRI would provide a non-invasive imaging technique that would improve the diagnosis and management of patients with acute myocarditis.

**Methods:**

Ten volunteers and 14 patients with suspected acute myocarditis underwent T2, T2* and late gadolinium enhancement (LGE) 3T MRI, with further T2* imaging at 24 hours after USPIO (ferumoxytol, 4 mg/kg) infusion, at baseline and 3 months. Myocardial oedema and USPIO enhancement were determined within areas of LGE as well as throughout the myocardium.

**Results:**

Myocarditis was confirmed in nine of the 14 suspected cases of myocarditis. There was greater myocardial oedema in regions of LGE in patients with myocarditis when compared with healthy volunteer myocardium (T2 value, 57.1±5.3 vs 46.7±1.6 ms, p<0.0001). There was no demonstrable difference in USPIO enhancement between patients and volunteers even within regions displaying LGE (change in R2*, 35.0±15.0 vs 37.2±9.6 s^−1^, p>0.05). Imaging after 3 months in patients with myocarditis revealed a reduction in volume of LGE, a reduction in oedema measures within regions displaying LGE and improvement in ejection fraction (mean −19.7 mL, 95% CI (−0.5 to −40.0)), −5.8 ms (−0.9 to −10.7) and +6% (0.5% to 11.5%), respectively, p<0.05 for all).

**Conclusion:**

In patients with acute myocarditis, USPIO-enhanced MRI does not provide additional clinically relevant information to LGE and T2 mapping MRI. This suggests that tissue-resident macrophages do not provide a substantial contribution to the myocardial inflammation in this condition.

Clinical trial registration NCT02319278; Results.

## Introduction

Acute myocarditis comprises a wide clinical spectrum from subclinical disease to severe heart failure and is a major cause of sudden death in young adults.^1^ Pathologically, it is characterised by inflammatory cell infiltration of the myocardium with evidence of myocyte necrosis that is not characteristic of an ischaemic aetiology.^2^ Causes of myocarditis include infections, immune-mediated injury and toxins (such as anthracyclines) although frequently no cause is identified.^3^

A variety of inflammatory cells infiltrate the myocardium during myocarditis. In viral myocarditis, the infiltrate is predominantly lymphocyte rich, but other cells including plasma cells, neutrophils, eosinophils, giant cells and macrophages are also present.^2 4–9^ Monocytes differentiate into macrophages at sites of myocarditis, suggesting that they play an important role in the injury or repair of the myocardium.^10^

Endomyocardial biopsy is considered the gold standard investigation for diagnosis. However, this is prone to sampling error and is uncommon in routine clinical practice.^3^ Cardiac MRI plays a key role in the diagnosis and shows typical appearances on T2-weighted and late gadolinium enhancement (LGE) imaging according to the Lake Louise Criteria.^11^ More recently, quantitative mapping techniques appear to improve diagnostic accuracy further.^12^

Iron oxide nanoparticles are generating interest as an MRI contrast agent able to detect macrophages, and clinical applications are now emerging.^13–21^ Ultrasmall superparamagnetic particles of iron oxide (USPIO) consist of an iron oxide core surrounded by a carbohydrate or polymer coating. They are small enough to extravasate passively through capillaries, where they are engulfed by tissue-resident macrophages^22^ and are detectable by T2*-weighted MRI. Thus, USPIO-enhanced MRI can identify tissue-resident macrophage activity and identify cellular inflammation within tissues.

In a promising preclinical study of autoimmune myocarditis,^23^ iron nanoparticles were ingested by inflammatory cells and improved distinction of areas with severe inflammation on MRI compared with conventional T2-weighted and gadolinium-enhanced MRI. We aimed to assess and quantify myocardial USPIO enhancement in acute myocarditis and to correlate enhancement with clinical measures of inflammation and oedema using T2 mapping and LGE MRI and compare these measures with healthy volunteers.^24^ We hypothesised that USPIO-enhanced MRI would detect myocardial macrophage activity in myocarditis and provide a cellular-specific non-invasive imaging technique that may aid and improve patient diagnosis and management.

## Methods

Patients were recruited from a single centre as part of an open-label observational multicentre cohort study (ClinicalTrials.gov (NCT02319278); Results). The study was performed in accordance with the Declaration of Helsinki, the approval of the Scotland A Research Ethics Committee and the written informed consent of all participants. The Medicines and Healthcare products Regulatory Authority of the UK gave Clinical Trial Authorisation for the study (EUDraCT 2013-002336-24).

### Study populations

Adult (>18 years of age) patients with suspected acute myocarditis were recruited into the study. The clinical diagnosis was made by an independent clinical cardiologist based on the history, ECG, serum troponin (ARCHITECT STAT troponin I assay, Abbott Laboratories, Illinois, USA) and other available imaging modalities. The diagnosis of myocarditis was verified from case note review by an independent cardiologist and retained for analysis if the MRI scan (reported and agreed by two independent radiologists) showed imaging features of myocarditis on LGE and/or T2 mapping.^11^ Healthy volunteers had no clinically significant medical history. Exclusion criteria were contraindication to MRI or ferumoxytol infusion, any other inflammatory comorbidity, renal failure (estimated glomerular filtration rate <30 mL/min/1.73 m^2^), pregnancy, breast feeding and women of childbearing potential without reliable contraception.

### Study protocol

Patients with suspected myocarditis and healthy volunteers underwent paired MRI scans at baseline, and patients were invited to return for repeat imaging after 3 months.

#### MRI

MRI was performed using a MAGNETOM Verio 3T MRI scanner running software V.VB17 (Siemens Healthcare, Erlangen, Germany), with a dedicated cardiac array coil. All images were acquired using ECG-gated breath holds. Routine steady state free precession (TrueFISP) sequences were used to acquire long-axis and short-axis images of the heart. Oedema imaging was conducted using a Siemens T2 mapping based on a prototype T2-prepared TrueFISP acquisition acquiring identical long-axis and short-axis slice positions. Quantitative USPIO imaging was performed in similar slice positions using a prototype T2*-weighted multigradient-echo acquisition using a volumetric shim applied over the entire heart volume. Standard cardiac slice widths (6 mm width with 4 mm gap) and eight echo times (2.1–17.1 ms range) with matrix size of 256×115 were acquired for T2* maps. The in-plane resolution differed as required for larger or smaller objects; generally, a field of view of 400×300 mm was used with an in-plane resolution of 2.6×1.6 mm. T2* relaxation times were calculated before and after administration of USPIO.

Immediately after the baseline T2, T2* and TrueFISP cine imaging, breath-held inversion recovery sequences in long-axis and short-axis planes were used to acquire late enhancement images following an intravenous administration of gadolinium contrast medium (0.1 mmol/kg; Gadovist, Bayer, Germany). Optimal inversion time (TI) was determined on a slice-by-slice basis using standard late-enhancement TI-scout protocols. The inversion-recovery late-enhancement short-axis slices were acquired using similar slice positions as the T2 and T2* imaging.

#### USPIO

Intravenous infusion of USPIO (ferumoxytol, 4 mg/kg; Rienso, Takeda Italia, Italy) was performed immediately following the baseline magnetic resonance scan over 15 min using a concentration of 2–8 mg/mL, diluted in 0.9% saline or 5% dextrose. Haemodynamic monitoring was conducted throughout.

### Image analysis

All T2*-weighted multigradient echo images for each patient were analysed using Circle CVI software (Circle CVI42, Canada). An experimentally determined threshold used in previous work^14^ for the coefficient of determination (r^2^>0.85) was used to exclude data that did not have an acceptable exponential decay when signal intensity (SI) was plotted against echo time. Individual images affected by artefact were excluded. The inverse of the mean T2* (R2*) for each regions of interest (ROI) was then calculated to assess the uptake of USPIO, where the higher the value, the greater the USPIO accumulation.

Analysis of T2 maps, LGE and ventricular volume and function was also performed using Circle CVI software. T2 and T2* data were collected immediately prior to USPIO administration. USPIO-enhanced T2* data were collected 24–25 hour following ferumoxytol administration.

#### ROI selection

ROI were drawn using the standard 16-segment cardiac model^25^ and panmyocardial values averaged using all 16 segments. As a final method of analysis to focus on inflamed myocardium, regions with contiguous LGE>1 cm^2^ on a single short-axis slice were retained and visually coaligned with corresponding T2 and T2* images. These corresponding coaligned regions were then averaged to form LGE*+*T2 and T2* regions.

### Statistical analysis

All statistical analysis was performed with GraphPad Prism V.6 (GraphPad Software, San Diego, California, USA). Shapiro-Wilk normality testing was carried out prior to testing. To compare USPIO uptake and myocardial oedema in patients and volunteers, R2* and T2 values were compared using paired and unpaired t-tests, Mann-Whitney and Wilcoxon tests depending on pairing and normality of data. To compare results at 3 months with baseline, a paired t-test was used. Statistical significance was defined as two-sided p<0.05.

## Results

Ten volunteers and 14 patients with suspected myocarditis were recruited. Nine patients had confirmed myocarditis according to imaging criteria and independent review. Baseline imaging was conducted within a week of diagnosis. Four patients had alternative diagnoses (takotsubo cardiomyopathy (n=2), lung cancer (n=1), polymyositis (n=1)) and one patient had an incidental finding of an unknown chest wall metallic implant. These five patients were excluded from further analysis. All nine patients retained for analysis had typical features of myocarditis on LGE imaging. Of these nine patients, one patient did not return at baseline for the 24 hours post-USPIO scan. Seven of the nine patients returned at 3 months for repeat imaging.

The healthy volunteer group was older with greater ejection fraction than the myocarditis group at baseline (p<0.001 for both, table 1). Patients with myocarditis had greater neutrophil count, C-reactive protein (CRP) and serum troponin concentrations than volunteers (p<0.05, p<0.01 and p<0.0001, respectively). There were no other significant differences between participant groups at baseline.

**Table 1:** Participant characteristics

	Healthy volunteers	Myocarditis (baseline)	Myocarditis (3 months)
Number	10	9	7
Female	6	1	1
Age (years)	50 (45–53)	28 (24–34)***	25 (23–35)
Body mass index (kg/m^2^)	26 (23–29)	25 (22–29)	25 (22–27)
Left ventricular ejection fraction (%)	61.1±4.1	51.0±4.9***	57.1±4.3
Late gadolinium enhancement (mL)	Nil	30.3±19.3	16.3±12.3
ECG	n/a	9/9	
ST elevation		7	
T-wave inversion		1	
Normal		1	
Coronary angiogram	n/a	4/9	
Normal		4	
Echo	n/a	8/9	
Wall motion abnormality		4	
Normal		4	
Blood tests			
White cell count (×10^9^/L)	5.8 (4.2–7.1)	7.1 (6.1–8.9)	5.1 (4.6–5.6)
Neutrophil count (×10^9^/L)	3.2 (2.2–4.2)	4.4 (3.7–5.2)*	2.8 (2.7–3.5)
Lymphocyte count (×10^9^/L)	1.7 (1.4–2.1)	1.8 (1.3–2.2)	1.8 (1.2–1.9)
Monocyte count (×10^9^/L)	0.4 (0.3–0.7)	0.6 (0.5–0.8)	0.4 (0.3–0.5)
C-reactive protein (mg/L)	1.0 (1.0–2.5)	38(14-63)**	2.0 (1.0–2.0)
Troponin (ng/L)	2.0 {1.5–4.0)	1 21 842 (3950–25 722)^****^	4.0 (2.0–6.0)

Mean±SD or median (IQR).

* p<0.05; **p<0.01; ***p<0.001; ****p<0.0001 (compared with volunteers at baseline).

Administration of ferumoxytol was well tolerated with no adverse reactions reported during or immediately after administration in any of the participants.

### USPIO enhancement

**Figure 1:**
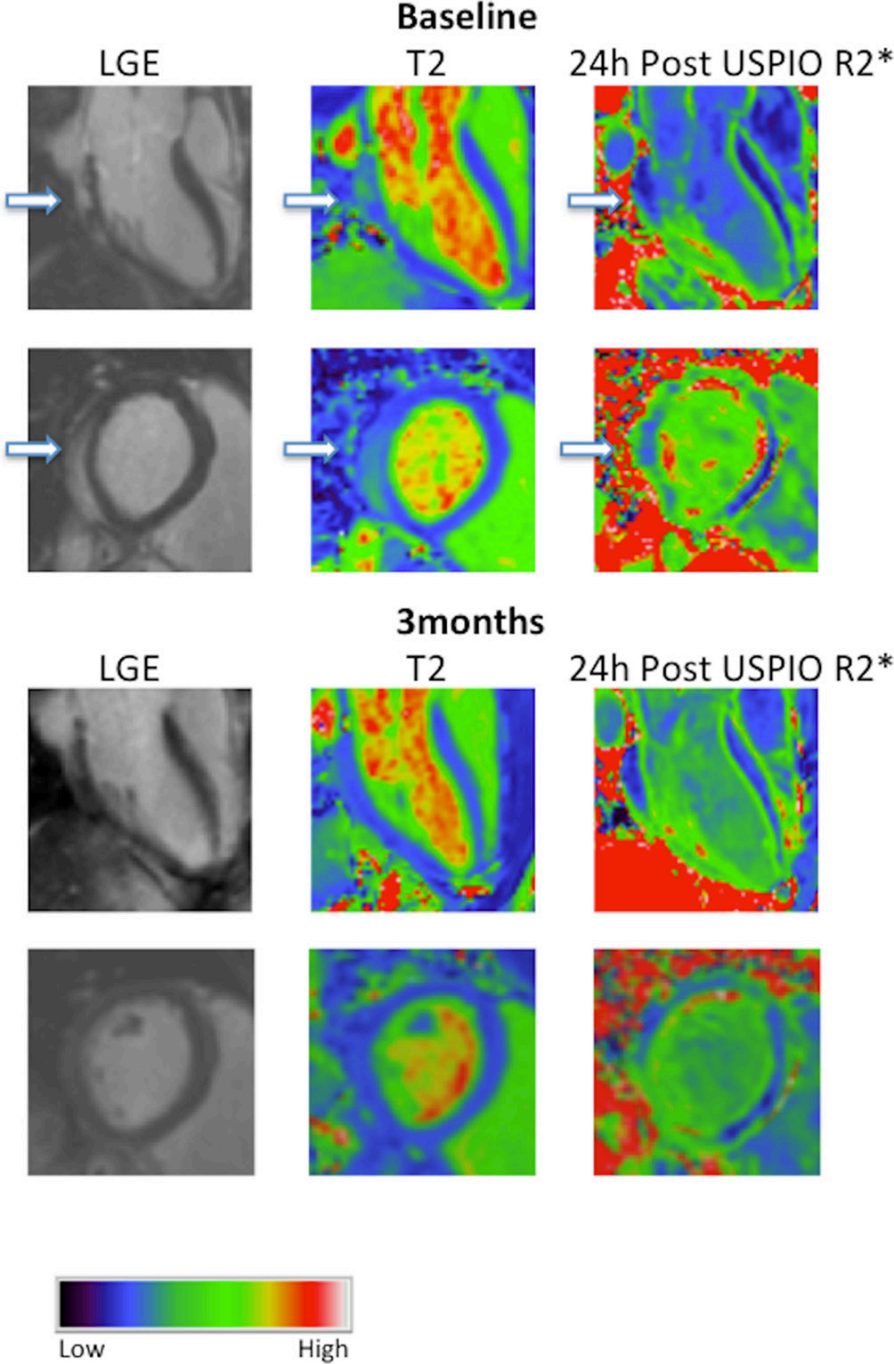
Images of patient with myocarditis. Three chamber and basal short-axis images of a patient with myocarditis displaying patchy posterolateral late gadolinium enhancement (LGE) (white regions, arrowed) that correspond to oedematous regions of myocardium on the T2 map (lighter regions, arrowed). There is no uptake of ultrasmall superparamagnetic particles of iron oxide (USPIO) in the post-USPIO R2* maps; corresponding regions may even have lower R2* in this patient. Myocardial oedema appears to normalise but subtle LGE remains at 3 months.

**Figure 2:**
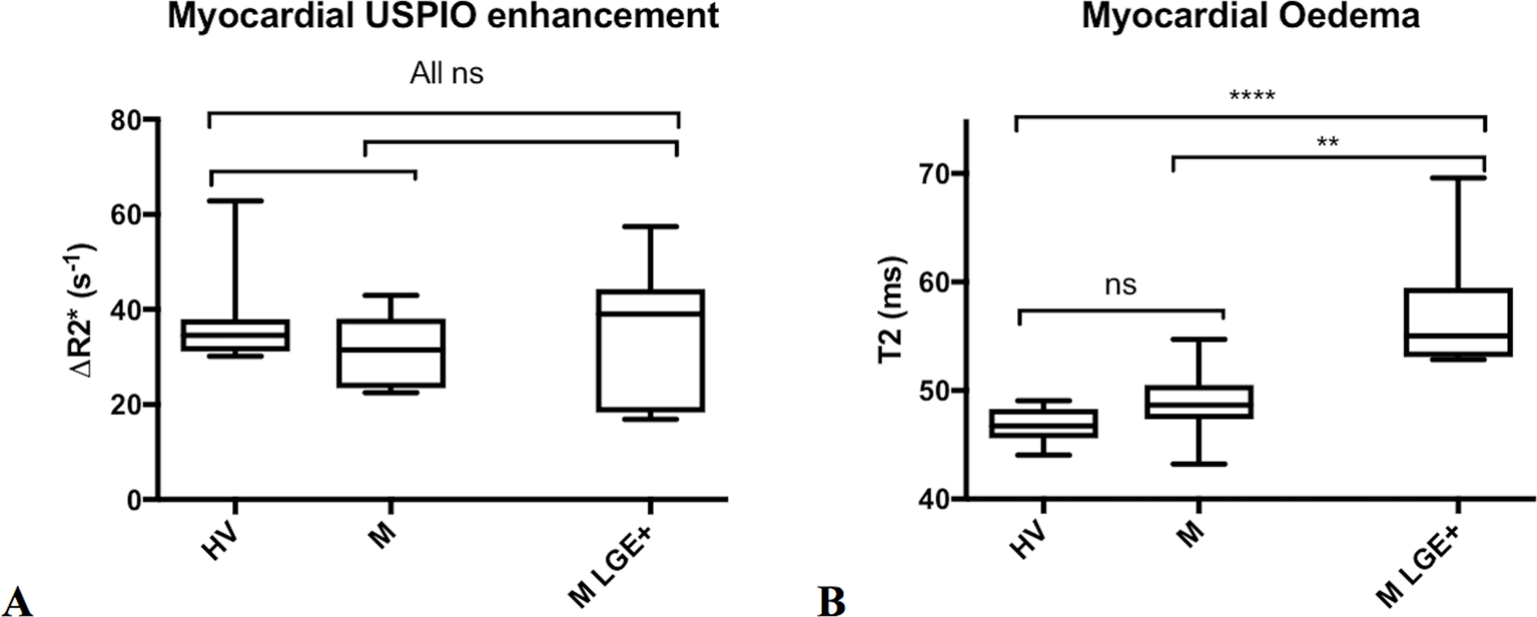
Myocardial USPIO enhancement vs Oedema at Baseline. Changes in myocardial R2* due to USPIO accumulation (A, left) and myocardial oedema by T2 mapping (B, right) are shown in healthy volunteers (HV) and patients with myocarditis (M) using panmyocardial average, and further limited to regions displaying LGE in patients with myocarditis (M LGE+). There were no significant differences in ΔR2* due to USPIO accumulation between all groups (p>0.05 for all). There was no difference in panmyocardial T2 between volunteers with patients with myocarditis. Myocardial regions displaying LGE (M LGE+) had greater T2 than the panmyocardial value for healthy volunteers and myocarditis patients (****p<0.0001 and **p<0.01 respectively).

At baseline, there were no differences in R2* values between healthy volunteers and patients with myocarditis (panmyocardium and LGE+ regions; Figures 1, 2). After USPIO administration, the R2* in all three groups increased but there was no difference in either the change in or post-USPIO, R2* between the groups (figure 2 and table 2).

**Table 2:** Myocardial R2* and T2 values in healthy volunteers and patients with myocarditis

	Healthy volunteers (panmyocardial)	Myocarditis (panmyocardial)	Myocarditis (LGE+ regions)
Panmyocardial pre-USPIO R2* (s^−1^)	46.9±4.1	43.7±5.3	51.8±8.3
Panmyocardial post-USPIO R2* (s^−1^)	84.2±12.4	75.6±11.3	86.1±15.3
Panmyocardial ΔR2* (s^−1^)	37.2±9.6	31.4±7.5	35.0±15.0
T2 (ms)	46.7±1.6	48.9±3.1	57.1±5.3****

Mean±SD.

****p<0.0001 (compared with volunteers).

### Myocardial oedema

There was no difference in panmyocardial T2 between volunteers and patients with myocarditis. Regions displaying LGE in myocarditis patients had higher T2 than panmyocardial values for both healthy volunteers and patients with myocarditis (p<0.0001 and p<0.01, respectively; figure 2 and table 2).

### Baseline versus 3-month imaging in patients with myocarditis

**Figure 3:**
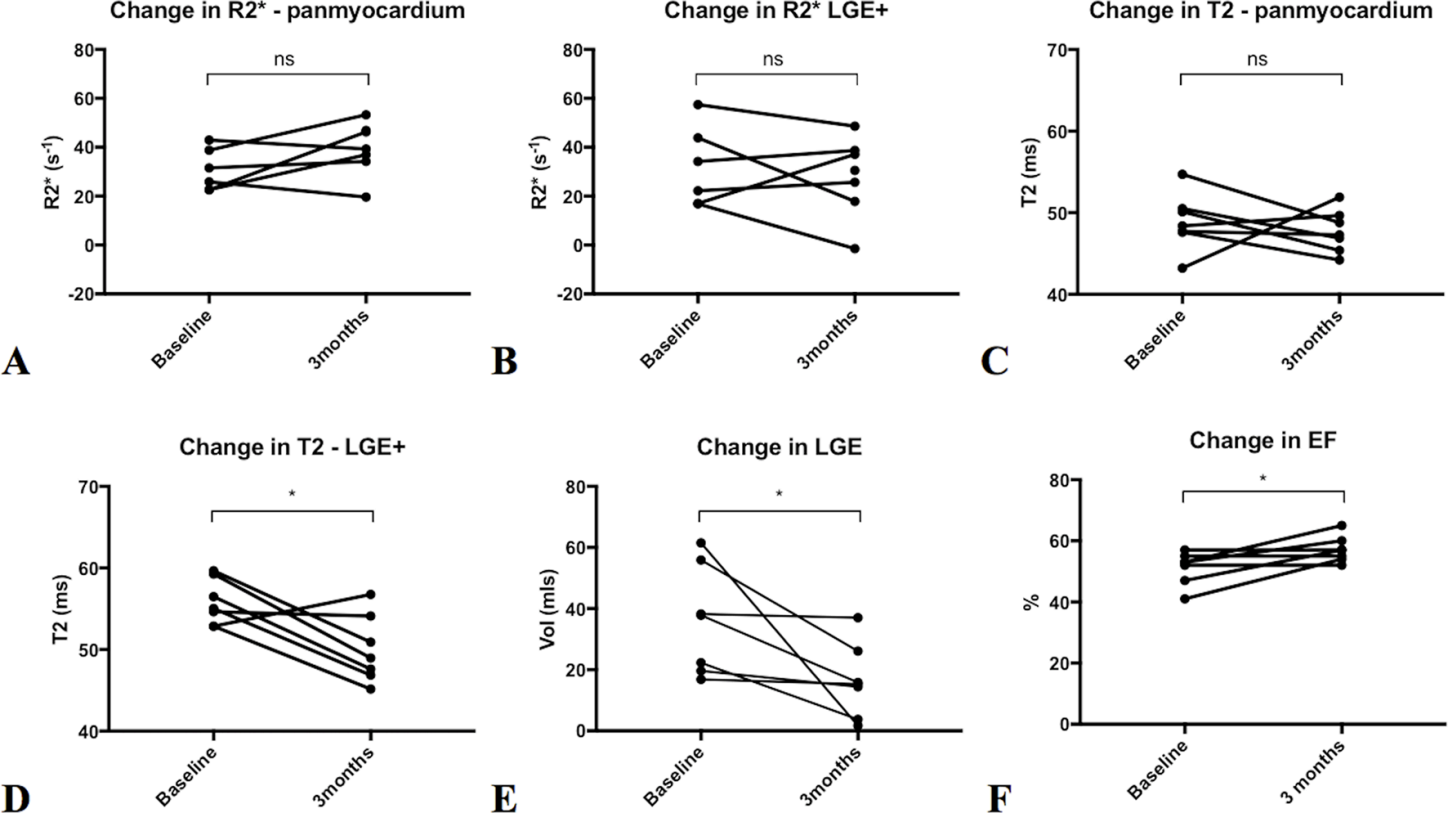
Changes on repeat imaging at 3 months in patients with myocarditis. There was no significant (ns) difference in ultrasmall superparamagnetic particles of iron oxide uptake between baseline and 3 months in both panmyocardium (A) and in regions displaying late gadolinium enhancement (LGE) (B). There was no difference in panmyocardial T2 (C) over 3 months. T2 in regions displaying LGE (D) reduced, as did the volume of LGE (E) over the 3-month period. There was an increase in ejection fraction (EF) between baseline and 3 months (F); *p<0.05.

There were no changes in myocardial USPIO uptake between baseline and 3 months either on panmyocardial analysis (mean +7.6 s^−1^ 95% CI (−4.8 to 19.9)) or in regions with LGE (−4.2 s^−1^ (−21.9 to 13.5), p>0.05 for both; figure 3). Panmyocardial T2 did not change over 3 months (−1.1 (-5.8 to 3.5), p>0.05). Volume of LGE and T2 within regions displaying LGE reduced over the 3-month period (−19.7 mL (−0.5 to −40.0) and −5.8 ms (−0.9 to −10.7), respectively, p<0.05 for both). Ejection fraction increased over the 3-month period (+6% (0.5 to 11.5), p<0.05).

## Discussion

For the first time, we report the combined assessment of myocarditis using LGE, T2 mapping and USPIO-enhanced T2* MRI. USPIO-enhanced T2* MRI has previously been used in man to assess cardiovascular inflammation in a range of different conditions,^14 16 21 26^ with preliminary positive results from a rodent model of myocarditis. This raised hope that USPIO-enhanced T2*MRI might add useful cell-specific clinical information in patients with myocarditis. However, while we found typical features of myocarditis using standard imaging with LGE and T2 mapping cardiovascular magnetic resonance (CMR), we did not detect USPIO enhancement within the myocardium of our patients. This suggests that USPIO-enhanced MRI provides no additional diagnostic value in these patients and that tissue-resident macrophages are not a major contributor to the cellular inflammation following acute myocarditis.

Our case study population of nine patients were excellent examples of myocarditis. They were young, with significantly elevated levels of systemic inflammatory markers, including neutrophil count, CRP and cardiac-specific high-sensitivity troponin, and all had typical features of myocarditis on CMR reported by two independent clinical radiologists. Most patients had ECG changes suggesting significant myocyte injury and several had normal coronary angiograms. All patients that did not have a coronary angiogram did not have any risk factors for coronary artery disease. We detected MRI features of myocarditis in every patient. LGE was discontinuous in nature and generally epicardial and subepicardial in distribution, but could be found in the midwall and occasionally in the subendocardium. LGE was never found in the subendocardium alone or confined to one coronary territory that would be more suggestive of myocardial infarction. In keeping with the typical distribution of myocarditis, most patients displayed LGE in the inferior, posterior and lateral walls. We are therefore confident that the diagnosis of myocarditis was robust in all patients, and an independent cardiologist verified this.

Myocardial oedema was easily visualised, and indeed quantified, using T2 mapping (Figure 2). We found intense myocardial oedema in our myocarditis cohort with maximal T2 values approaching 70 ms on T2 mapping. Within regions displaying LGE (LGE+), there was a profound increase in T2 when compared with volunteers. This signal was so powerful that a trend remained evident when comparing panmyocardial T2 values to those of volunteers, although the difference was not statistically significant. Seven patients returned for repeat imaging assessment at 3 months, where we recorded an improvement in clinical features: a reduction in the volume of LGE (by around 50%), a reduction in oedema within inflamed (LGE+) regions and an overall improvement in ejection fraction.

We were unable to detect USPIO accumulation in our cohort of patients with myocarditis. We know that macrophages can be present in myocarditis, although they are generally not the predominant cell type. It is possible that patients within our group had predominantly lymphocytic or neutrophil-rich cell infiltrate and the finding of greater levels of neutrophils in patients with myocarditis at baseline may support the latter. The clinical care team did not feel that endomyocardial biopsy was justified in any of the cases, and lack of histological data is clearly a limitation. In recent work, we have detected USPIO-laden macrophages in patients with recent myocardial infarction and demonstrated macrophage uptake of USPIOs in biopsies taken at the time of cardiac surgery.^13^ We therefore acknowledge that while some USPIO-laden macrophages may have been present in patients with myocarditis, there were either insufficient numbers of macrophages or USPIO engulfment was inhibited or deficient. Ultimately, USPIO-enhanced T2* CMR was unable to demonstrate increased macrophage activity and lacked sensitivity for detecting myocarditis.

A further contributory reason for failure to detect USPIO enhancement within inflamed regions may be due to difficulties encountered in USPIO-enhanced T2* imaging. These are discussed in greater detail elsewhere,^24^ but artefact due to breathing and in particular, ‘blooming’ artefacts from the vascular organs of the lungs, liver and stomach can be problematic. This can make analysis more challenging by having to exclude the affected later echo times from T2* decay curve fitting. Indeed, ‘blooming’ artefacts from nearby tissues and organs most commonly affect the inferior and lateral walls, which is the usual site of inflammation in myocarditis. Therefore, the myocardial regions of greatest interest were often the most difficult to analyse although this can usually be overcome.^24^ This may go some way to explain why there is greater spread of R2* data in patients with myocarditis, especially in LGE+ regions. Finally, patients with myocarditis often had symptoms of pleurisy and pericarditis, and despite adequate analgesia, often found the long breath holding required for T2* imaging difficult.

Some final limitations also deserve mention. First, the sample size was small, but numbers in this pilot study were sufficient to show that USPIO-enhanced T2* CMR fails to add clinically relevant information to CMR imaging parameters of LGE and T2 mapping for individual patients. Second, the control volunteer group was not age or sex matched but we have no reason to believe that this influenced the results. Lastly, myocardial geometry is affected by several factors that vary between scans such as the presence of tissue oedema, heart rate and blood volume status and accurate coregistration can be challenging.

Despite these results, USPIO-enhanced imaging still holds promise as a non-invasive imaging method for the diagnosis and monitoring of tissue inflammatory macrophages in the heart. We have recently found that USPIO-enhanced MRI can detect and serially monitor macrophages after myocardial infarction (MI)^13^ and work is under way at our centre as part of this project (NCT02319278) to assess the value of this technique in diagnosing cardiac sarcoidosis and cardiac transplant rejection. If successful, USPIO-enhanced MRI may provide a platform on which to assess existing and novel therapeutic interventions that might modify the inflammatory process, not only after MI but also in other inflammatory processes affecting the heart.

In conclusion, we have shown that in patients with acute myocarditis, USPIO-enhanced T2* MRI does not provide additional clinically relevant information to that of LGE and T2 mapping MRI. This suggests that tissue-resident macrophages do not provide a substantial contribution to the myocardial inflammation in this condition.

**Key messages**

**What is already known about this subject?**

Ultrasmall superparamagnetic particles of iron oxide (USPIO) are ingested by tissue macrophages that can be visualised using MRI to highlight areas of inflammation within the heart.

**What does this study add?**

USPIO-enhanced T2* MRI does not provide additional clinically relevant information to that of LGE and T2 mapping MRI. This suggests that tissue-resident macrophages do not provide a substantial contribution to the myocardial inflammation in this condition.

**How might this impact on clinical practice?**

USPIO-enhanced T2* MRI may still prove to be of value in diagnosing and monitoring conditions with macrophage-driven myocardial inflammation, but its role in assessing acute myocarditis appears limited.
